# Pregestational overweight and obesity are associated with differences in gut microbiota composition and systemic inflammation in the third trimester

**DOI:** 10.1371/journal.pone.0200305

**Published:** 2018-07-13

**Authors:** María Florencia Zacarías, María Carmen Collado, Carlos Gómez-Gallego, Heini Flinck, Janne Aittoniemi, Erika Isolauri, Seppo Salminen

**Affiliations:** 1 Functional Foods Forum, Faculty of Medicine, University of Turku, Turku, Finland; 2 Institute of Agrochemistry and Food Technology, National Research Council (IATA-CSIC), Department of Biotechnology, Valencia, Spain; 3 Department of Clinical Microbiology, Fimlab Laboratories, Tampere, Finland; 4 Department of Pediatrics and Adolescent Medicine, Turku University Hospital, Turku, Finland; 5 Department of Clinical Sciences, Faculty of Medicine, University of Turku, Turku, Finland; University College London, UNITED KINGDOM

## Abstract

The obesity epidemic is a global challenge, and the velocity of propagation is high in the population at reproductive age. Overweight and obesity during pregnancy have been associated with high birth weight and an increased risk of childhood obesity, reinforcing the risk of other non-communicable diseases. Obesity involves chronic low-grade systemic inflammation. New biomarkers for early detection of obesity risk are urgently required. The aim of this study was to identify the connection between pregestational BMI (pre-BMI) status and inflammatory biomarkers during the third trimester of pregnancy and their association with intestinal microbiota composition. Fifty-four pregnant women were classified according to pre-pregnancy BMI as normoweight, overweight, or obese. Weight gain, inflammatory biomarkers (hs_CRP, haptoglobin, and suPAR), and microbiota composition were assessed during the third trimester. A significant lower weight gain for obese mothers and a positive correlation between pre-BMI and inflammatory biomarkers were detected (Spearman’s correlation). Haptoglobin levels were significantly higher in overweight and obese mothers. Higher Firmicutes levels and a higher ratio Firmicutes/Bacteroidetes were observed in the overweight and obese subjects. High hs_CRP and haptoglobin levels were also correlated with decreased microbiota diversity (Shannon index), whereas haptoglobin and hs_CRP values were correlated with several microbiota components, such as *Ruminococcus gnavus* and *Faecalibacterium*, and with specific phyla in the normoweight and overweight mothers; no significant associations with microbiota were found for suPAR. In conclusion, haptoglobin and hs_CRP reflected pregestational BMI status and related microbiota components, but haptoglobin was a better biomarker for microbiota associated with overweight. suPAR was associated with low grade inflammation dependent on pre-pregnancy BMI, but it was not related to deviated microbiota profiles.

## Introduction

Profound alterations occur during normal human gestation, including inflammatory changes [1]. An increased risk for adverse pregnancy outcomes for both the mother and the offspring has been associated with pregestational body mass index (BMI) and also with elevated inflammation [2,3]. Several inflammatory biomarkers have been studied in association with potential complications during pregnancy. C-reactive protein (CRP) is the most extensively examined inflammatory marker associated with adverse events, and significantly higher values have been observed in obese mothers [4]. Changes in plasmatic haptoglobin have been proposed as a biomarker of preeclampsia [5–7]. The soluble urokinase plasminogen activating receptor (suPAR) is a newly characterized inflammatory marker during pregnancy, but thus far, no clear evidence of its predictive value for the later development of pregnancy complications has been found [8,9]. Inflammatory changes during pregnancy are accompanied by shifts in gut microbiota. Specific alterations occur during pregnancy, particularly during the third trimester, favoring a pro-inflammatory status, with a decline in butyrate-producing bacteria and an increase in Actinobacteria and Proteobacteria, although these changes need to be further identified [10,11,12]. Maternal pregestational BMI and weight gain over gestation have been related to some of these specific gut microbial shifts [13,14]. Obesity is associated with low-grade inflammation [15] and causally related to insulin resistance and dysbiosis, a shift in gut microbiota. As occurs in obesity, pregnancy is associated with the production of pro-inflammatory factors due to increased adipose tissue, especially in the third trimester. The metabolic changes, exaggerated in overweight and obese women, also affect microbiota composition, which is characterized by reduced bacterial richness and activity of the microbiota.

Increased CRP and haptoglobin levels are indicators of inflammation observed in human obesity [16]. Circulating high-sensitive CRP (hs_CRP) is a widely accepted marker of chronic low-grade systemic inflammation [17], and it has been associated with heart disease [18], colorectal cancer [19], and complications of diabetes and obesity [20]. Haptoglobin is a plasma glycoprotein strongly associated with diseases that have inflammatory causes [21]. In obesity, its expression is dramatically increased [22], and a potential role of haptoglobin as a macrophage chemoattractant in adipose tissue has been established [23]. suPAR is considered a biomarker of both inflammation and immune activation since it is released from several cell types in response to inflammatory stimulation [24], and higher levels can be associated with a risk of cardiovascular disease (CVD), type II diabetes, cancer, and premature mortality, independent of CRP levels [25]. Higher levels of the three inflammatory biomarkers-CRP, haptoglobin, and suPAR-have been found in obese subjects, particularly in children and adolescents [17,26,27]. Gut microbiota is connected to energy homeostasis and inflammation and can be considered as a factor in the pathophysiology of obesity [28]. Metagenomic data have allowed for the identification of changes in the composition and metabolic function of the gut microbiota in obese subjects [29]. The Firmicutes-Bacteroidetes ratio may play a role in the development of obesity, although results are also contradictory [30]. As for the link between obesity, inflammatory biomarkers, and microbiota composition, some correlations have been established in children [28], adolescents [29], and adult subjects [31–35]. However, no associations between inflammatory markers and microbiota changes during pregnancy have been reported.

In this study, we focused on pregnant women to assess the connection between pregestational BMI status and gut microbiota composition and systemic inflammation during the third trimester of pregnancy.

## Material and methods

### Study design and subjects

Subjects for the study were selected from a prospective follow-up study of 256 women [36]. Written informed consent was obtained from the participants, and the study protocol was approved by the Ethics Committee of the Hospital District of South-West Finland (registration number NCT00167700). Women were eligible if they had no metabolic or chronic diseases such as diabetes and did not consume probiotics nor received dietary counselling during pregnancy. Altogether, 54 women fulfilled the criteria for the present study and were grouped according to their pre-pregnancy BMI (pre-BMI; kg/m^2^) (Table 1). No underweight women were part of the study. To obtain pre-BMI, height was measured at the first visit and the pre-pregnancy weight was obtained from well-women clinic documents in which the self-reported pre-pregnancy weight is recorded during the first visit. Gestational weight gain (GWG) was calculated by subtracting self-reported pre-pregnancy weight from that recorded at the third trimester prenatal visit. Birth data on the infants were obtained from hospital records.

**Table 1:** Maternal and pregnancy clinical characteristics.

	p value	Normal weight	Overweight	Obese
Age (years)	0.6667	29.62±4.20	30.44±3.60	29.62±2.33
Weight before pregnancy (kg)	<0.0001	61.68±7.06*	73.17±6.26*	93.09±11.16*
Height (m)	0.2354	1.66±0.05	1.64±0.06	1.68±0.08
Pre-BMI (kg/m^2^)	<0.0001	22.31±1.58*	27.16±1.37*	32.99±2.83*
Gestational weight gain (kg)	0.0013	9.94±3.73	9.81±2.31	5.55±3.62*
Gestational age (wk)	0.4979	39.8±1.5	40.2±1.8	40.4±1.3
Birth weight of the child (kg)	0.3986	3.69±0.38	3.59±0.0.57	3.83±0.37
Vaginal deliveries (%)	0.1651	92	72	91

Data are mean ± SD or percentage (for vaginal deliveries). ANOVA test with Tukey's post hoc test for multiple comparisons or a Chi-square test (for vaginal deliveries) were used to determine the statistical differences.

*Significant difference (p<0.05) from the rest of the groups.

### Samples

#### Blood and fecal samples

Fecal samples were collected for analysis of gut microbiota composition in the third trimester of pregnancy (30–35 wk of gestation). Serum samples were collected at the same time with fecal samples and stored at -80°C.

#### Biomarker methods

Biomarkers were determined in the third trimester. The serum suPAR level was determined using a commercial enzyme immunoassay (suPARnostic^®^ AUTO Flex ELISA, ViroGates A/S, Birkerød, Denmark). Serum haptoglobin and hs_CRP levels were determined by immunonephelometry using the BN ProSpec^®^ System (N Antiserum to Human Haptoglobin and CardioPhase^®^ hsCRP, Siemens Healthcare Diagnostics Products GmbH, Marburg, Germany).

#### DNA extraction and 16S bacterial gene sequencing

Total DNA was isolated from fecal samples using a commercially available kit, InviMag® Stool DNA kit (Invitek GmbH, Berlin, Germany), in the KingFisher magnetic particle processor (Thermo Electron, Vantaa, Finland) as described previously [37]. Purified DNA was determined using a Qubit® 2.0 Fluorometer (Life Technology, Carlsbad, CA, USA) and normalized to 5 ng/μL for amplicon libraries. The V3-V4 region of the 16S rDNA gene was amplified by PCR following Illumina protocols. After 16S rDNA gene amplification, the multiplexing step was performed using a Nextera XT Index Kit (Illumina, San Diego, CA, USA). One microliter of the PCR product was checked with a Bioanalyzer DNA 1000 chip (Agilent Technologies, Santa Clara, CA, USA) and libraries were prepared using a 2x300pb paired-end run (MiSeq Reagent kit v3) according to manufacturer’s instructions (Illumina) at FISABIO sequencing service (Valencia, Spain).

### Statistical analysis

All clinical data from individual participants were combined according to their pre-BMI in normoweight (N/W), overweight (O/W), and obese (O/O) groups. Normal distribution of the data was assessed with the Shapiro Wilk test. An ANOVA test with Tukey's post hoc test for multiple comparisons and a Chi square test for comparison of vaginal deliveries, were used to determine the statistical differences among the groups. Biomarker differences were tested between N/W and O/W or O/O groups with a Mann–Whitney U-test (non-normal distributions). Associations between pre-BMI or GWG and biomarkers were computed with a Spearman Rho Test correlation. P-values ≤0.05 were regarded as statistically significant. Analysis were performed using GraphPad Prism version 5.00 for Windows (GraphPad Software, San Diego, California, USA).

### Bioinformatics

Sequencing data were processed as described previously [38], and sequenced management was performed using the QIIME pipeline (version 1.9.0) [39]. Chimeric sequences and sequences that could not be aligned were also removed from the data set. An open reference OTU picking method using a 97% identity to the Greengenes 13_8 database was selected. OTUs with a relative frequency below 0.01 were removed. Sequences not classified at the domain level, or classified as Cyanobacteria and Chloroplasts, were removed from the dataset. Calypso software version 8.20 (http://cgenome.net/calypso/) was used for microbiota statistical analysis and for Spearman Rho-test correlations involving an abundance of bacterial taxa. Microbiota data was normalized with total sum normalization (TSS) combined with square root transformation before the statistical analysis. P-values ≤0.05 were regarded as statistically significant.

## Results

### Clinical characteristics and inflammatory markers

**Fig 1:**
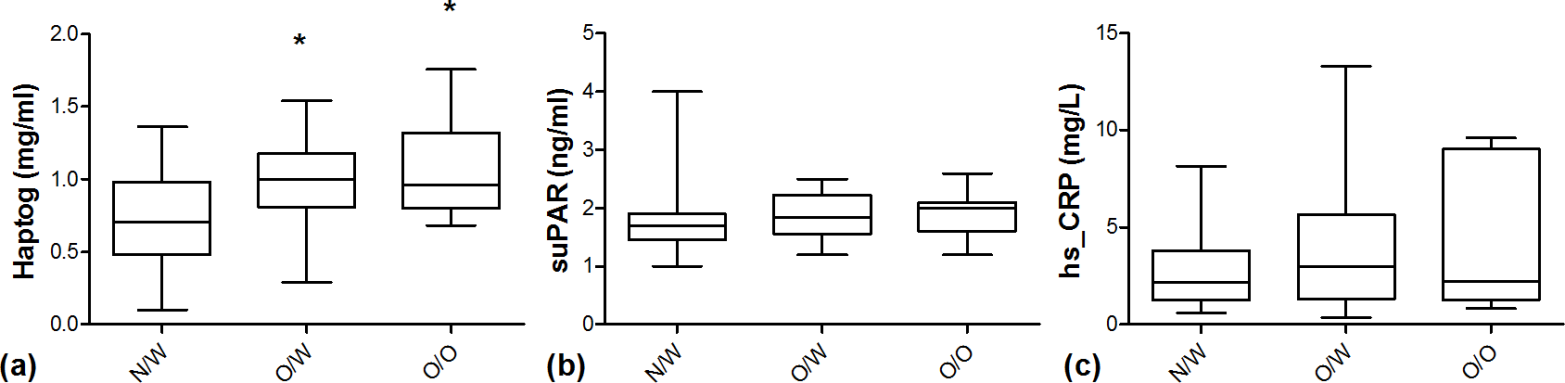
Serum levels of inflammatory biomarkers in pregnant women at third trimester. (a) Haptoglobin (Haptog); (b) soluble urokinase plasminogen activator receptor (suPAR), and (c) high sensitive C-reactive protein (hsCRP) levels in normoweight (N/W), overweight (O/W), and obese (O/O) pregnant women. *Significant differences compared to N/W with p value < 0.05 (Mann Whitney U test).

**Fig 2:**
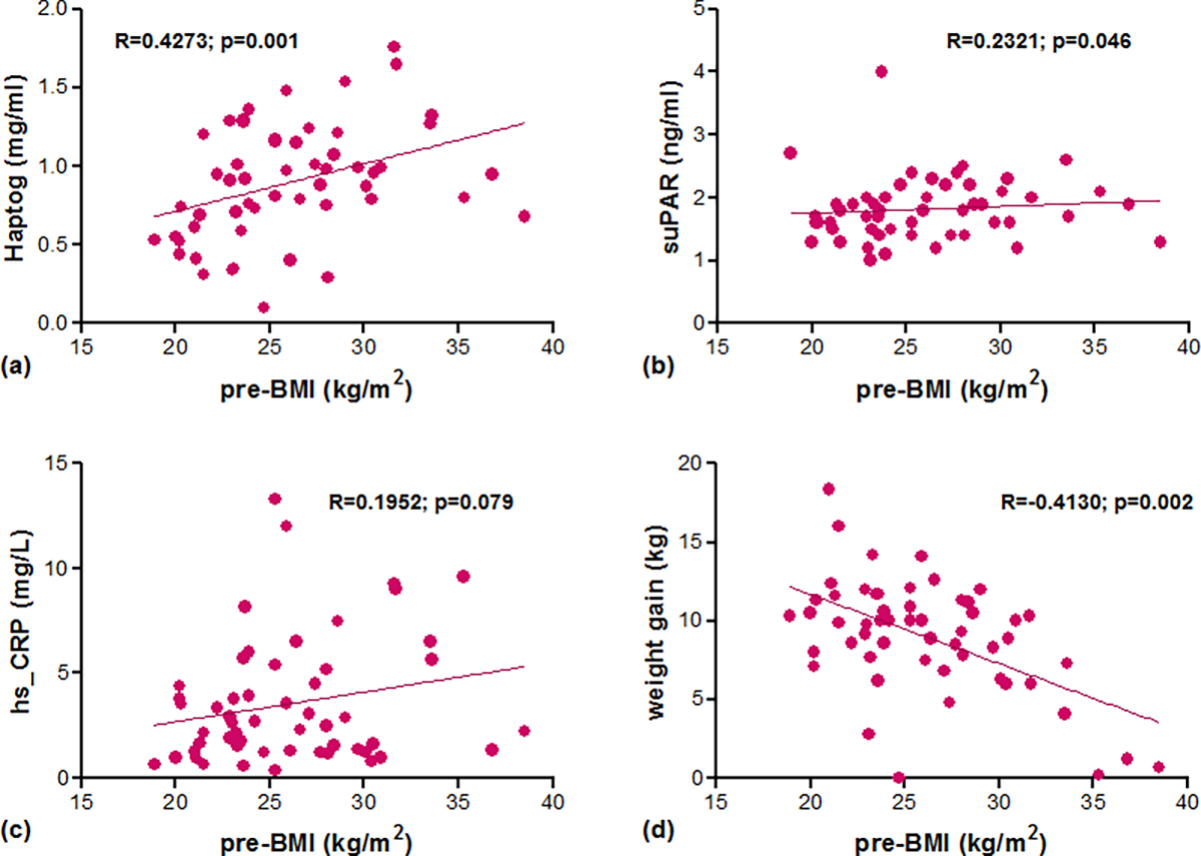
Associations between pre-BMI and inflammatory biomarkers and weigh gain at third trimester. Spearman's rank correlation coefficient (Rho) between pregestational BMI (pre-BMI) and (a) haptoglobin, (b) suPAR, (c) hs_CRP, and (d) weight gain at third trimester.

The characteristics of the pregnant women included in this study are presented in Table 1. Fifty-four women were classified according to their pre-BMI as normoweight (BMI 18.5–24.9 kg/m^2^; n = 25), overweight (BMI 25–29.9 kg/m^2^; n = 18), and obese (BMI ≥ 30 kg/m^2^; n = 11). Besides the significant differences for pre-BMI between groups, only a significant lower gestational weight gain was observed for obese mothers (Table 1). Serum biomarkers were assessed during the last trimester of pregnancy. Only haptoglobin levels were significantly higher in overweight and obese mothers compared to normal weight ones (Fig 1). When overweight and obese mothers were grouped together, higher haptoglobin and suPAR levels compared to normoweight mothers were observed, whereas only a trend to be increased was observed for hs_CRP (results not shown). The three biomarker candidates-haptoglobin, suPAR, and hs_CRP-correlated positively with pre-BMI (p = 0.001, p = 0.046, and p = 0.079, respectively; Spearman’s correlation). Also, an inverse relationship between pre-BMI and GWG (Fig 2) but no correlation of GWG with any of the inflammatory biomarkers was observed.

### Microbiota and biomarkers in the third trimester are associated with pregestational BMI

**Fig 3:**
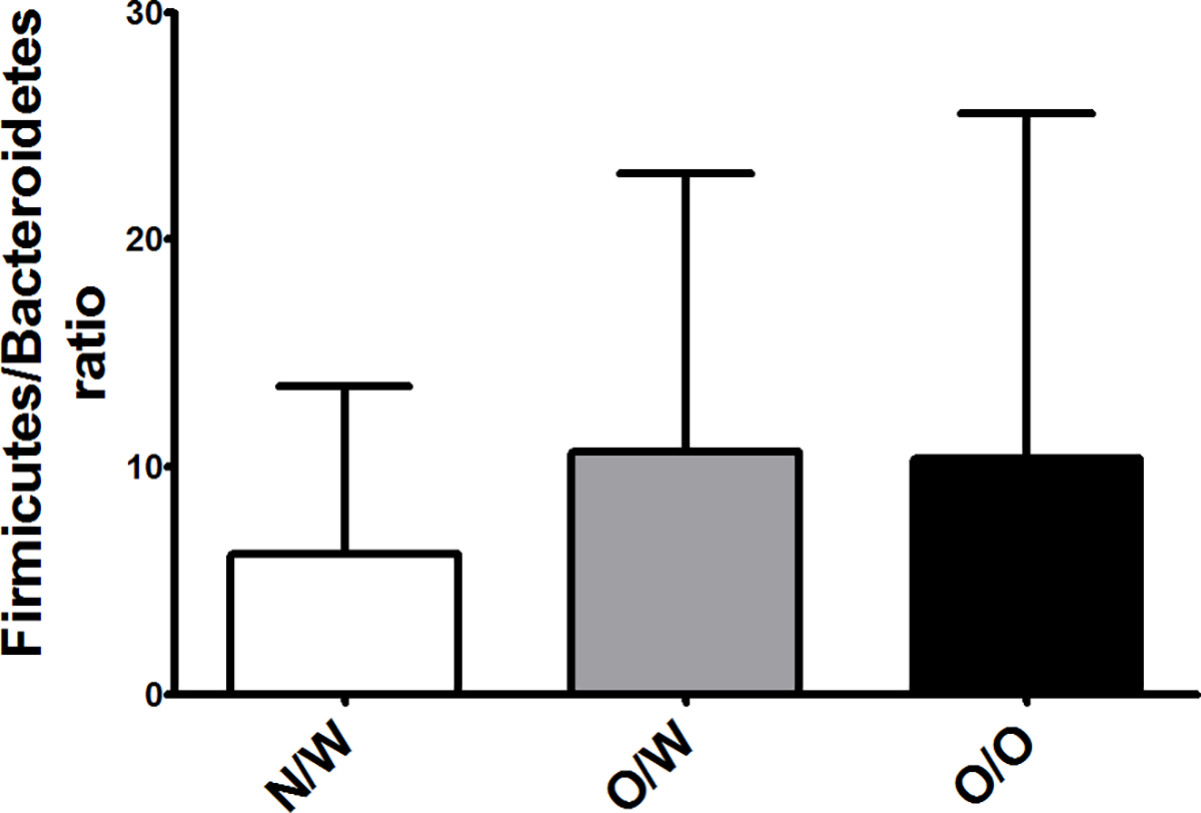
Firmicutes/Bacteroidetes ratio in gut microbiota at third trimester of pregnancy. Normoweight (N/W), overweight (O/W), and obese (O/O) mothers. *Significant differences compared to N/W with p value < 0.05 (Mann Whitney U test).

Fecal microbiota composition was associated with pre-BMI. Significantly different abundances of phylum Firmicutes (p = 0.028; Table 2) were observed, with higher levels in overweight and obese mothers compared to normal weight ones (S1 Fig, t test). When the Firmicutes/Bacteroidetes ratio was evaluated, higher but non-significant values were observed in the overweight (p = 0.0597) and obese groups (p = 0.0741) (Fig 3), and only when overweight and obese mothers were grouped together was the ratio F/B statistically significantly higher (p = 0.0252) (results not shown). Differences at the family and genus level according to pre-BMI status were also detected (Table 2), with higher Lachnospiraceae and Actinomycetaceae for obese mothers, lower Bacteroidaceae for overweight mothers, and lower Desulfovibrionaceae in obese and overweight mothers compared to normal weight ones (S1 Fig, t test).

**Table 2:** Relative abundance of bacterial taxa at various taxonomic levels in association with pre-BMI status.

**Taxa (phylum)**	**p value**	**Normal weight**	**overweight**	**obese**
Firmicutes	0.028*	8.34	8.73	8.82
Tenericutes	0.045*	0.15	0.06	0.07
Bacteroidetes	0.064	2.49	1.70	1.69
Proteobacteria	0.14	0.71	0.35	0.39
Verrucomicrobia	0.31	1.43	1.86	1.19
Synergistetes	0.34	0.07	0.00	0.00
TM7	0.46	0.09	0.08	0.10
Actinobacteria	0.51	3.99	3.59	3.79
**Taxa (family)**				
Lachnospiraceae	0.0032*	5.66	6.4	6.81
Leuconostocaceae	0.021*	0.056	0.14	0.098
Desulfovibrionaceae	0.022*	0.13	0.038	0.016
Actinomycetaceae	0.029*	0.18	0.19	0.25
Bacteroidaceae	0.034*	2.18	1.38	1.45
**Taxa (genus)**				
*Dialister*	0.0098*	0.4	0.2	0.64
*Faecalibacterium*	0.01*	0.96	1.5	0.99
*Coprococcus*	0.013*	2.61	3.04	3.54
*Catenibacterium*	0.016*	0.0012	0	0.25
*Actinomyces*	0.018*	0.17	0.19	0.25
*Blautia*	0.032*	3.2	3.55	4.04
*Bacteroides*	0.034*	2.18	1.38	1.45

Data are mean values.

*The significance of difference between the groups was assessed (p<0.05; ANOVA).

**Fig 4:**
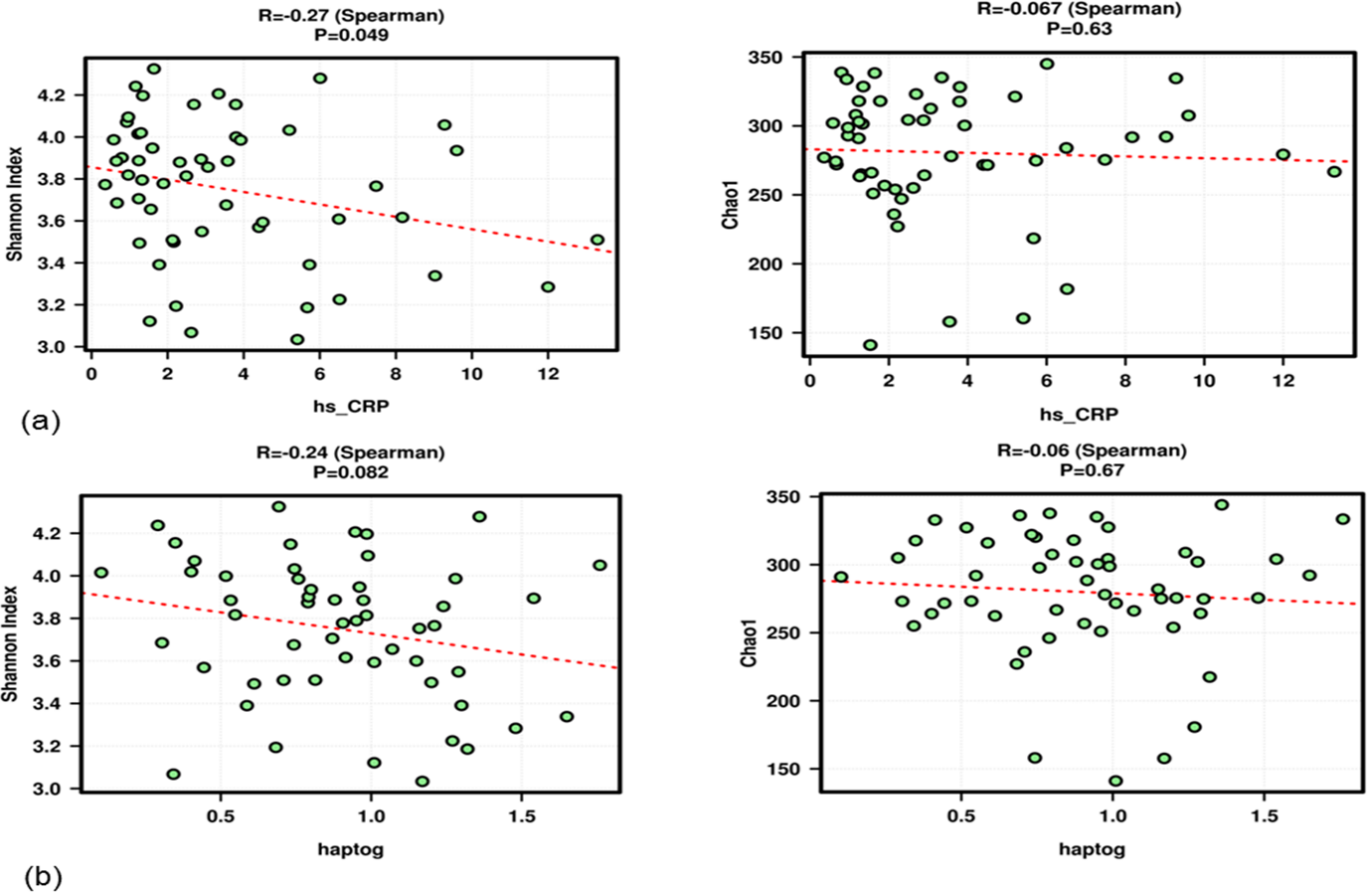
Associations between inflammatory biomarkers with microbiota diversity (Shannon index) and richness (Chao1 index). Spearman's rank correlation coefficient (Rho) for (a) hs_CRP and (b) haptoglobin. No associations were found for suPAR.

Associations between the inflammatory biomarkers and microbiota composition were studied. An alpha diversity analysis was performed and data generated for the Chao1 and Shannon indexes. High hs_CRP (Fig 4(A) and haptoglobin (Fig 4(B) levels were correlated with a decreased diversity (Shannon index) but not with modifications in microbiota richness (Chao index). The hs_CRP and haptoglobin values were also correlated with several microbiota components at the genus and species level (Table 3). No associations between suPAR and microbiota diversity, richness, or composition were found.

**Table 3:** Correlations between the relative abundance of bacterial taxa at various taxonomic levels and maternal biomarkers.

Biomarker	taxa	p value	Rho	Mean abundance^ϕ^	Positive Samples
**hs_CRP**	*Lactococcus*	0.006*	-0.37	0.29	50
	*Unclassified Bifidobacterium*	0.026*	-0.30	0.82	48
	*Lachnobacterium*	0.043*	-0.28	0.13	35
	*Faecalibacterium prausnitzii*	0.044*	-0.27	1. 1	54
	*Unclassified Coriobacteriaceae*	0.048*	-0.27	0.95	53
**Haptog**	*Unclassified Bacteroides*	0.021*	-0.31	1.40	54
	*Ruminococcus gnavus*	0.025*	0.31	0.42	54
	*Unclassified Erysipelotrichaceae*	0.030*	0.29	1.20	54
	*Bifidobacterium longum*	0.047*	0.27	1.90	54
	*Unclassified Coriobacteriaceae*	0.051	-0.27	0.95	53

Rho is Spearman correlation coefficient for each bacterial taxa.

^ϕ^Square root transformed data.

*Significant correlations with p value < 0.05.

No associations were found for suPAR.

**Fig 5:**
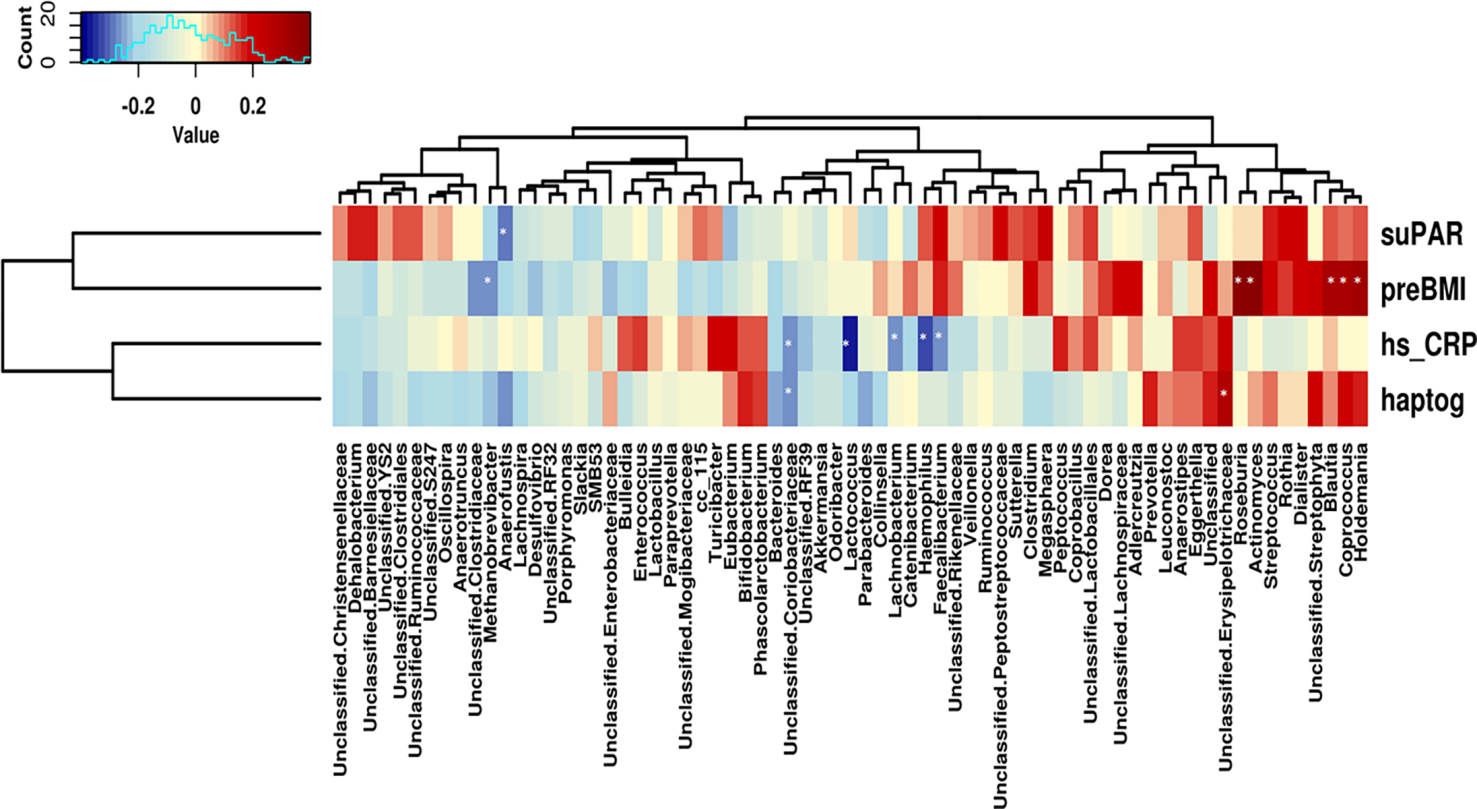
Associations between microbiome and serum biomarkers and pregestational BMI. Heatmap generated from Spearman rank test correlation analysis shows associations between hs_CRP, haptog, suPAR and pre-BMI and abundance (Square root transformed data) of specific bacterial genera. Red to blue scale: positive to negative associations.

When the link between microbiota and biomarkers was evaluated separately for each pre-BMI group, haptoglobin was the only inflammatory biomarker associated with specific phyla (S2 Fig). In normoweight women, a positive correlation with Proteobacteria and a negative correlation with Firmicutes (R = 0.50; p = 0.011 and R = -0.44; p = 0.028, respectively; Spearman correlation) were detected, whereas hs_CRP levels tended to be positively associated with Actinobacteria (R = 0.35; p = 0.087; Spearman correlation). In overweight mothers, haptoglobin showed a strong negative association with Bacteroides and also with Verrucomicrobia (R = -0.67; p = 0.0028 and R = -0.51; p = 0.031, respectively; Spearman correlation) and a positive association with Firmicutes (R = 0.53; p = 0.023; Spearman correlation). No significant associations between biomarkers and specific phyla were found for obese mothers. Finally, in order to assess the relationships between specific bacterial groups, pre-BMI, and biomarkers, a Spearman correlation analysis was performed, showing a potential link between gut microbiota composition and these parameters (Fig 5). The results showed a core of positively correlated bacteria with pre-BMI, haptoglobin, and hs_CRP, including some members of the Erysipelotrichaceae (*Holdemania)* and Lachnospiraceae (*Coprococcus* and *Blautia*) family, and a few negative associations, mainly *Bacteroides*, Coriobacteriaceae, and *Methanobrevibacter*. Negative correlations between hs_CRP and specific bacteria were also found, particularly other members from Lachnospiraceae and *Faecalibacterium*, whereas haptoglobin was associated positively with Enterobacteriaceae. SuPAR values were not correlated with microbiota diversity, richness, or composition (results not shown).

## Discussion

Our study is the first to assess the association of maternal pregestational BMI with inflammatory microbiota composition during the third trimester of pregnancy. In this work, the inflammatory biomarkers haptoglobin and suPAR during the third trimester positively correlated with pregestational BMI. A positive correlation trend was also observed for hs_CRP.

Haptoglobin has been associated with adiposity and metabolic syndrome, and a predictive role for type 2 diabetes, dependent on BMI, has also been reported [40]. In pregnant women, its role as a diagnostic marker of preeclampsia (PE) and of fetal and maternal outcomes was evaluated, but even if lower haptoglobin levels were associated with PE occurrence, it was not possible to define it as biomarker for pregnancy complications [41]. Based on our results, haptoglobin was the most efficient at reflecting overweight or obese status before pregnancy.

Although a trend towards higher hs_CRP levels was observed in this study for overweight and obese mothers grouped together, it was not statistically significant. Previous studies have focused on the behavior of CRP during pregnancy, but results have not been conclusive. Belo *et al*. [1] found no significant changes throughout gestation, with great inter-subject variations during pregnancy, even though median values were consistently higher throughout pregnancy, compared to non-pregnant women. Other studies found a significant decrease in serum CRP levels from early to late pregnancy, although other inflammatory markers, such as IL-6 and TNF-α, were increased [42,43]. Furthermore, Friis *et al*. [44] reported the effect of pregnancy on adiposity-related inflammation and concluded that while normal pregnancy exhibits some proinflammatory features, no additive effect was observed on the BMI-dependent inflammation at the end of pregnancy. However, higher CRP levels in obese mothers were reported by Stewart *et al*. [43], and a trend toward being higher was also observed in the work by Christian and Porter [42].

In our study, pre-BMI was also inversely correlated with GWG during pregnancy, but no correlation between GWG and biomarkers could be established. These results are consistent with those previously reported by Friis *et al*. [44], who found no association between weight gain and changes in the circulatory levels of inflammatory markers. In Collado *et al*. [13], no GWG differences between obese and normoweight mothers were detected, although a trend toward being lower was detected in the obese subjects in the third trimester. In a recent study, Broskey *et al*. [45] reported the influence of total GWG on neonatal outcomes and the effect of the timing of it. Their results showed a significant inverse relationship between pre-BMI and total GWG, and obese mothers gained the least amount of total weight in early and late pregnancy when compared with overweight and normoweight mothers.

Gut microbiota is not only involved in nutrient absorption, digestion, and metabolic activities, but it has also been linked with energy efficacy and storage [11]. Limited information is available regarding the effect of obesity on the shifts in microbiota composition during pregnancy [46]. The results of this study show how maternal BMI prior to pregnancy affects microbiota composition, with a higher relative abundance of Firmicutes and a trend toward a higher Firmicutes/Bacteroidetes ratio in the third trimester for overweight and obese mothers. This could imply that overweight/obesity-related dysbiosis was already present and that this state was not significantly altered throughout pregnancy. Collado *et al*. [13] reported a positive correlation between *Bacteroides* and weight gain during pregnancy. This could explain the tendency toward lower *Bacteroides* levels in the obese mothers found in our study, considering a significant lower GWG was found for this group. A previous study on pregnant women also reported a trend toward higher Firmicutes abundance in obese rather than overweight women at 16 weeks’ gestation, but the absence of a normoweight group made it difficult to be conclusive about the relationship between microbiome and maternal pre-BMI [46]. In our study, no differences in Firmicutes levels were observed between overweight and obese mothers, but significantly higher abundances were observed in both groups compared to normoweight mothers. Associations between pre-BMI and specific bacterial taxa were assessed. At the family level, higher Lachnospiraceae levels were linked to higher pre-BMI. At the genus level, some significant differences were detected: higher *Coprococcus*, *Catenibacterium*, *Actimomyces*, and *Blautia* abundances were found in the obese group and lower *Bacteroides* and higher *Faecalibacterium* levels were found in the overweight group compared to the normal weight group. These results are in agreement with previous studies. A higher prevalence of Lachnospiraceae has been associated with obesity [47] and metabolic disorders [48], and a positive correlation between this family and maternal BMI was also detected for pregnant women at 16 weeks’ gestation [46]. As for other microbial components, an obese microbiota characterized by *Blautia* and *Coprococcus* has been reported in Japanese adults by Kasai *et al*. [49].

When the link between α-diversity and inflammatory biomarkers was studied, lower diversity, evidenced by Shannon index, was associated with higher values of hs_CRP and haptoglobin. Lower diversity in obese subjects was previously reported [50,51], including in pregnant women [10]. Some studies also associated diversity and richness with inflammatory status. Verdam *et al*. [52] have linked reduced diversity with a more inflammatory profile in obese subjects, which is in agreement with our findings. Adverse metabolic conditions, including adiposity and a more pronounced inflammatory phenotype, was found by Cotillard *et al*. to be related to lower richness [53]. The association between biomarkers and specific bacterial taxa was also established. Haptoglobin was the most efficient biomarker for detecting differences between groups, specifically reflecting a negative correlation with Firmicutes abundance in normoweight mothers and significant positive and negative associations with Firmicutes and Bacteroidetes, respectively, in the overweight group. At the species level, a positive correlation between haptoglobin and *Ruminococcus gnavus* was observed. Positive associations between haptoglobin and *Ruminococcus* or *Clostridium* cluster XIVa, from which *Ruminococcus gnavus* is a member, have been previously described [54,55]. For hs_CRP, a significant inverse correlation with *Faecalibacterium* was found, which is in agreement with results reported by Martínez *et al*. [33]. In general, hs_CRP and haptoglobin, together with pre-BMI, are positively associated with members of the related families Erysipelotrichaceae (*Holdemania*, *Bulleidia*) and Lachnospiraceae (*Blautia*, *Coprococcus*, *Anaerostipes*) and are negatively associated with Coriobacteriaceae, *Bacteroides*, and *Methanobrevibacter*, the main representative of the human gut Archea. These results confirm previously reported bacterial associations with BMI and obesity status. The predominance of Prevotellaceae (phylum Bacteroidetes) and Erysipelotrichaceae and Lachnospiraceae (phylum Firmicutes) has been positively associated with BMI [49,51,56]. Reduced levels of *Desulfovibrio*, *Bacteroides*, and *Methanobrevibacter* have been reported in obese subjects [30,34,35,57]. Few studies have reported differences in microbiota composition during pregnancy and the influence of pre-BMI and weight gain [13,46,58]. Recent studies have revealed the importance of the maternal microbial environment in infants’ health both early and later in life, suggesting that the transfer of the altered gut microbiota from obese pregnant women to their infants may lead to metabolic disorders [59,60]. Maternal obesity has been associated with adverse outcomes, such as an increased risk of metabolic syndrome during childhood [61]. Considering that pregnancy and obesity share common characteristics, such as a state of lower grade inflammation and shifts in microbiota composition, the evaluation of these factors and their association is of great value. One major limitation of this study is that the number of subjects included was limited. Despite this limitation, our study provides insight into the association between pregestational BMI and microbiota composition during the third trimester, adding the levels of inflammatory biomarkers as a factor. Outlining these associations could be the first step in defining whether the changes observed in the third trimester can be measured by the selected biomarkers and whether they are predictive for offspring outcomes, specifically overweight or obesity risk. Future studies including a larger number of subjects should be performed. The pre-BMI microbiome biomarker associations in the present study were assessed in pregnant women in the third trimester only. To definitively establish the relationship between these factors, a cohort of control (non-pregnant) women and measurements during the stages of early pregnancy should be also investigated.

## Conclusions

Pre-pregnancy BMI is linked to the inflammatory status of the mothers and their intestinal microbiota composition, in the third trimester of pregnancy. Taken together, haptoglobin was found to detect overweight/obese status and was related to proinflammatory microbiota components. Higher haptoglobin and CRP levels suggested a decrease in microbiota diversity. In our study population, suPAR confirmed the low grade of inflammation of obesity, but its effect was not reflected in the microbiota profile. Obesity is associated with microbial dysbiosis as well as systemic inflammation. The use of inflammatory biomarkers as potential tools for predicting pregnancy or offspring outcomes is of interest but further studies including non-pregnant women as part of the study population should be performed.

## Supporting information

S1 Fig **Relative abundance of bacterial taxa at (a) phylum, (b) family and (c) genus level, in association with pre-BMI status.** Differences in microbiota composition between normal weight (red), overweight (blue) and obese (green) groups. * Significantly different (p<0.05; t test).
(TIF)

S2 Fig Heatmap microbiota-biomarkers depending on pre-BMI status. *Significant differences in microbiota composition at phylum level (p<0.05; Spearman correlation).
(TIF)
