# Supplementary figures and images for: Pregestational overweight and obesity are associated with differences in gut microbiota composition and systemic inflammation in the third trimester
Source: PLoS One. 2018 Jul 13;13(7):e0200305. doi: 10.1371/journal.pone.0200305 (PMC6044541; doi:10.1371/journal.pone.0200305)

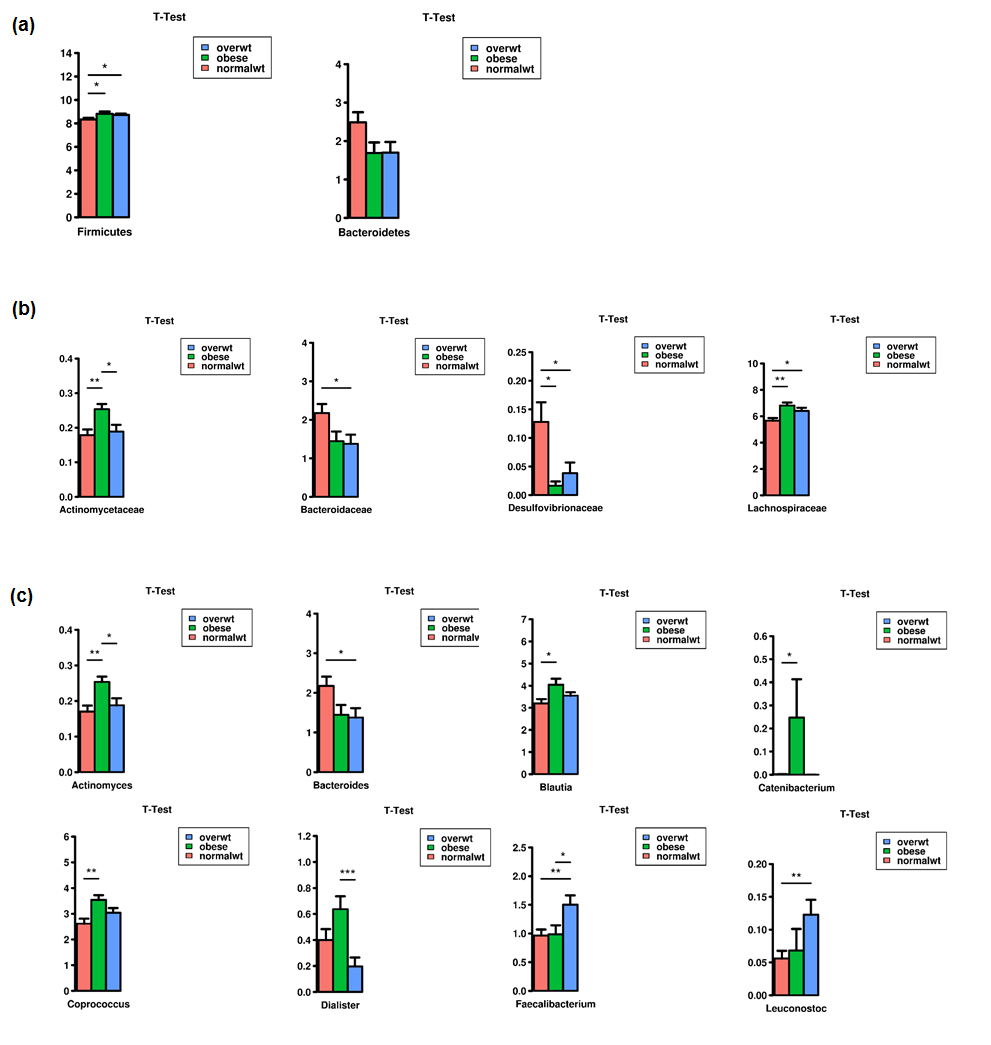

Supplement: S1 Fig — Relative abundance of bacterial taxa at (a) phylum, (b) family and (c) genus level, in association with pre-BMI status. Differences in microbiota composition between normal weight (red), overweight (blue) and obese (green) groups. * Significantly different (p<0.05; t test). (TIF) [file pone.0200305.s001.tif]

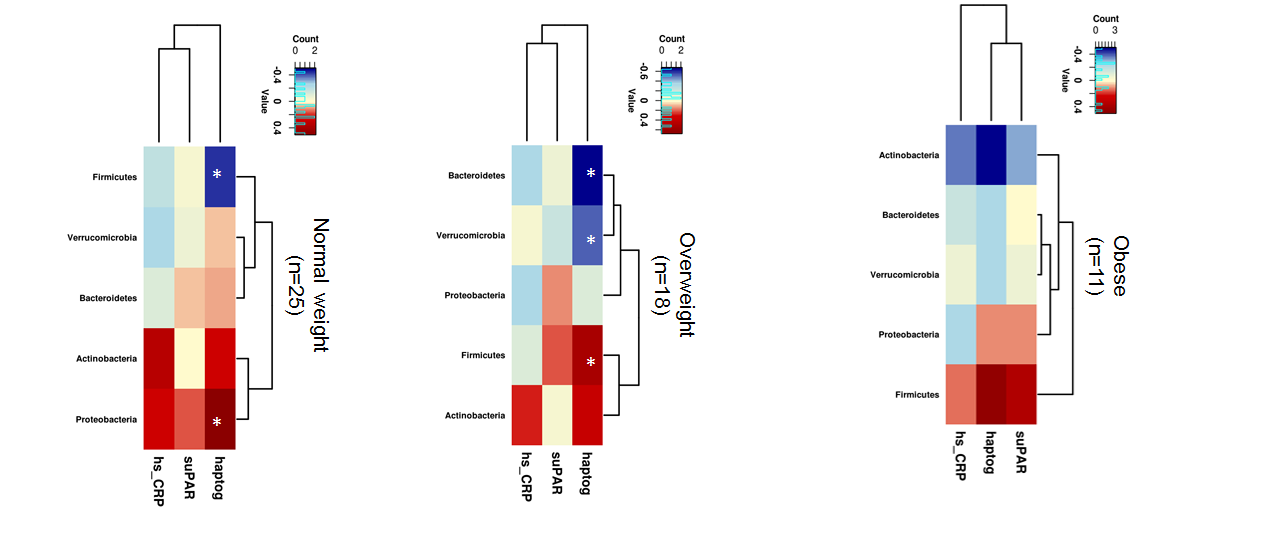

Supplement: S2 Fig — *Significant differences in microbiota composition at phylum level (p<0.05; Spearman correlation). (TIF) [file pone.0200305.s002.tif]
